# Spontaneous atlanto-axial dislocation and trisomy 21: causal factors and management

**DOI:** 10.11604/pamj.2019.33.3.18109

**Published:** 2019-05-06

**Authors:** Fah Bouaré, Mohamed Lmejjati, Davis Mpando

**Affiliations:** 1 Department of Neurosurgery, Mohammed VI Hospital, Cadi Ayyad University, Marrakesh, Morocco

**Keywords:** Atlanto-axial joint dislocation (C1-C2), trisomy 21, Down syndrome, spinal cord compression, neurosurgery

## Abstract

Spontaneous atlanto-axial (C1-C2) dislocation is an atlanto-axial instability, found in 10 to 30% of trisomy 21 patients, the majority of whom is asymptomatic. We report a case of a 21 years-old woman, with trisomy 21, admitted in our department presenting a spinal cord compression syndrome with right hemiparesis associated with a cervicalgia evolving for 3 months of admission without trauma. Standard cervical radiography showed a C1-C2 dislocation with posterior displacement of the odontoid process. A cervical computerized tomography revealed a C1-C2 dislocation with significant recoil of the odontoid process. A cervical magnetic resonance imaging (MRI) confirmed the bulbo-medullar junction compression on the dislocation. The surgical treatment consisted of a cervico-occipital fixation. The laxity of the transverse ligament is one of the main causes of C1-C2 dislocation; hypoplasia, malformation or complete absence of the odontoid process; are also predisposing factors. It must be early detected. The treatment of choice is surgical also by arthrodesis of C1 to C4 + graft and enlargement of the occipital foramen or occipito-cervical arthrodesis by synthetic graft and Cotrel-Dubousset system or occipito-C4 arthrodesis + laminectomy of C1 and enlargement of the occipital foramen.

## Introduction

Spontaneous atlanto-axial (C1-C2) dislocation is an atlanto-axial instability found in 10 to 30% of trisomic 21 patients, most of whom is asymptomatic [1, 2]. Only 1-2% of people with downs develop symptoms [2]. The aim of this work is to specify the etiological factors and the surgical treatment of spontaneous C1-C2 dislocation in trisomy 21.

## Patient and observation

**Figure 1:**
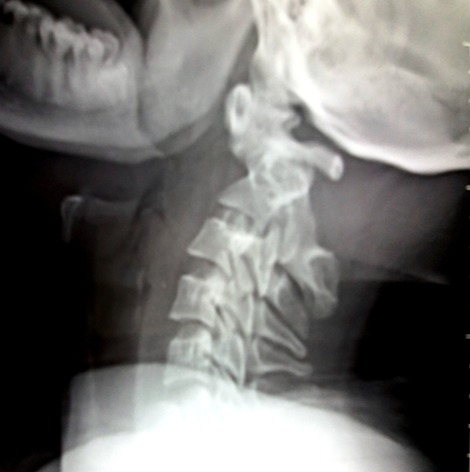
Cervical spine X-ray showing C1-C2 dislocation with posterior displacement of the odontoid process

**Figure 2:**
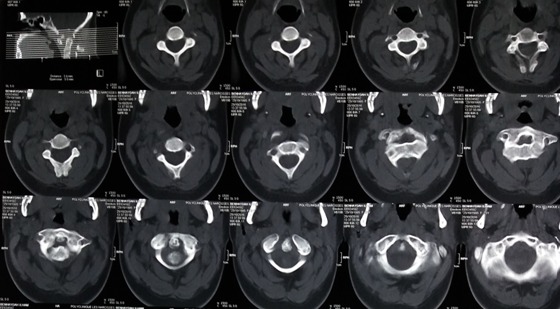
Cervical CT in axial section bone window showing C1-C2 dislocation with significant recoil of the odontoid process

**Figure 3:**
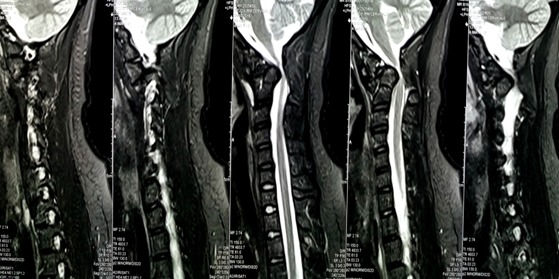
Cervical spinal MRI showing C1-C2 dislocation with compression of the bulbo-medullary junction

**Figure 4:**
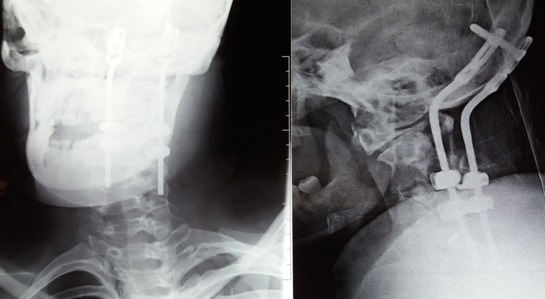
Post-operative X-Ray after occipito-cervical fixation

A 21-years-old woman with a trisomy 21, admitted in our department with a spinal cord compression syndrome made of cervical spinal syndrome and right hemiparesis evolving for 3 months without context of trauma. Standard cervical radiography (Figure 1) showed a C1-C2 dislocation with posterior displacement of the odontoid process. A cervical tomodensitometry (CT) in axial section of the bony window (Figure 2) confirmed the dislocation C1-C2 with a significant recoil of the odontoid process. A cervical magnetic resonance imaging (MRI) (Figure 3) was made, which found C1-C2 dislocation with compression of the bulbo-medullary junction. A surgical decision was made regarding the neurological repercussions of dislocation. The goal of the surgical treatment was to reduce, decompress and fix by a posterior approach. The patient was in prone position, the head on the Mayfield head holder, and an occipito-cervical incision from the external occipital protuberance to C4 was performed. Under X-ray, we put a Vertex type material (medtronic) which allowed the placement of 2 occipital screws and 4 hooks on all laminae of C2 and C3. Postoperative radiography was performed (Figure 4). The clinical evolution was marked by a slight recovery of muscle strengths on the right hemi-body and an infection of the area of the operative incision which was treated by application of antiseptics, healing products and oral antibiotics.

## Discussion

**Figure 5:**
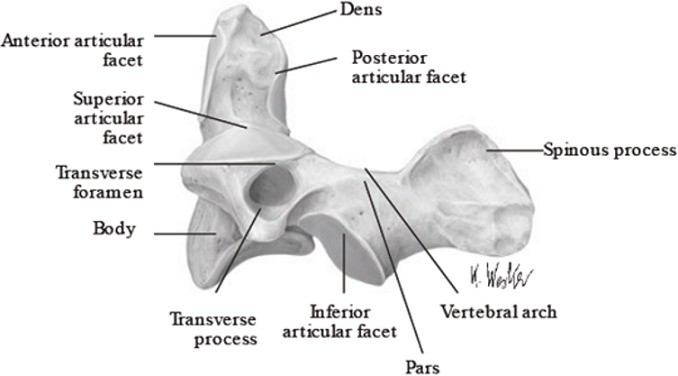
View of the C2 odontoid process

**Figure 6:**
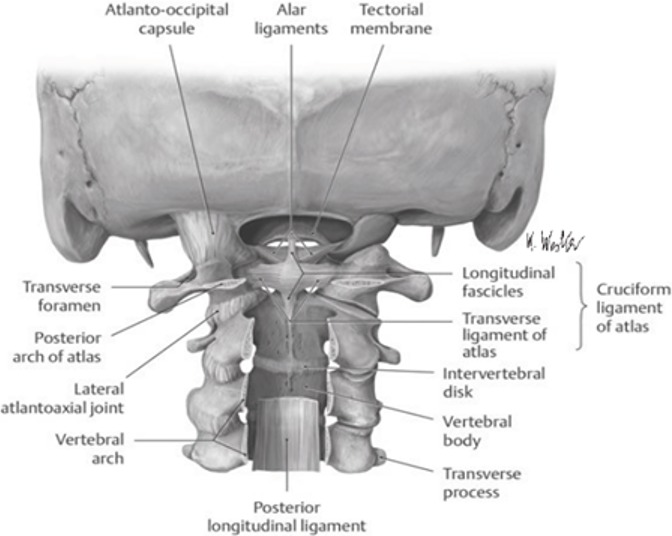
View of the different ligaments attaching the C2 odontoid process to C1 and occiput

As anatomic review, the C2 odontoid process or the dens, visible on the Figure 5, has many attachment ligaments on C1 and occiput. Among these ligaments we can see the transverse ligament on the Figure 6 [3]. The C1-C2 dislocation or instability in trisomy 21 may be present in the neonatal period [4]. The laxity of the transverse ligament is one of its main causes; hypoplasia, malformation or complete absence of the odontoid process is also predisposing factors [5]. Screening is done by careful clinical examination (looking for signs of spinal cord compression) and standard radiography with the flexion and hyper-extension of the neck. This screening is recommended twice, between the age of 5 and 10 and at the age of 15 [6]. The dislocation can lead to spinal cord compression and should be investigated by dynamic radiographies in case of trisomy 21 [4]. Its signs may be: torticollis, spastic hemiparesis, quadriparesis, anal incontinence, neurogenic bladder, paresthesia; or ataxia, disturbances of equilibrium [7].

Imaging (CT scan, MRI) can be used to diagnose, in addition to C1-C2 dislocation, odontoid bone dysplasia and cervical spinal cord compression associated with signs of myelomalacia [7]. Surgical treatment by posterior approach may consist of an arthrodesis of C1 to C4 + graft and enlargement of the occipital foramen or occipito-cervical arthrodesis by synthetic graft and Cotrel-dubousset system or occipito-C4 arthrodesis + laminectomy of C1 and enlargement of the occipital foramen [7]. The choice of occipito-cervical fixation by the vertex type material (medtronic) is that it allows a reduction with decompression and effective stabilization of the reduction. The predominant risk is a spinal cord compression during hyper-extension of the neck, especially during intubation preparation [5]. Its surgical treatment allows a favorable evolution except for completely deficit patients [7].

## Conclusion

Spontaneous atlanto-axoid dislocation in the trisomy 21 patient may be congenital, related to transverse ligament laxity, hypoplasia, malformation, or complete absence of the odontoid process. Its screening must be done carefully, clinically and radiologically. It must be early diagnosed and the treatment of choice is surgery.

## Competing interests

The authors declare no competing interests.

## References

[1] Davidson MA (2008). Primary care for children and adolescents with Down syndrome. Pediatr Clin North Am.

[2] Chaanine A, Hugonenq C, Lena G, Mancini J (2008). Les complications neurologiques liées à la trisomie 21. Arch Pediatr.

[3] Hobbs Jonathan, Ramos Edwin, Baaj Ali A, Mummaneni Praveen V, Uribe Juan S, Vaccaro Alexander R, Greenberg Mark S (2016). Craniovertebral Junction. Handbook of spine surgery.

[4] Lefvre Y, Rigal J, Mariey R, Durbec-Vinay A (2012). Anomalies rachidiennes diagnostiquées en période néonatale - Conduite à tenir. Archives de Pédiatrie.

[5] (1994). Neurologic sequelae secondary to atlantoaxial instability in Down syndrome - Implications in otolaryngologic surgery. Arch Otolaryngol Head Neck Surg.

[6] Cullen S, O'Connell E, Blake NS, Ward OC (1989). Atlantoaxial instability in Down’s syndrome: clinical and radiological screening. Ir Med J.

[7] Alvarez N, Kao A, Schneck MJ, Talavera F (2011). Atlantoaxial instability in Down syndrome.

